# Seasonal variability of the vitamin D effect on physical fitness in adolescents

**DOI:** 10.1038/s41598-020-80511-x

**Published:** 2021-01-08

**Authors:** Gregorio P. Milani, Giacomo D. Simonetti, Valeria Edefonti, Sebastiano A. G. Lava, Carlo Agostoni, Maurus Curti, Andreas Stettbacher, Mario G. Bianchetti, Franco Muggli

**Affiliations:** 1 Istituto Pediatrico della Svizzera Italiana, 6500 Bellinzona, Switzerland; 2 Pediatric Unit, Fondazione IRCCS Ca’ Granda Ospedale Maggiore Policlinico, via della Commenda 9, 20122 Milan, Italy; 3 Department of Clinical Sciences and Community Health, Università degli Studi di Milano, 20122 Milan, Italy; 4 Università della Svizzera Italiana, 6600 Lugano, Switzerland; 5 Pediatric Cardiology Unit, Department of Pediatrics, Centre Hospitalier Universitaire Vaudois (CHUV), University of Lausanne, 1011 Lausanne, Switzerland; 6 Viollier AG, 4123 Allschwil, Switzerland; 7 Swiss Federal Department of Defence, 3010 Bern, Switzerland

**Keywords:** Paediatric research, Paediatrics

## Abstract

Studies investigating the relationship between vitamin D and physical fitness in youth have provided inconsistent findings. Recent evidence indicates that the expression of receptors and vitamin D-modulated genes in young subjects has a seasonal profile. Therefore, we investigated the role of vitamin D on physical fitness across seasons in a total of 977 male adolescents. Anthropometrics, lifestyle, dietary habits, biochemical profiles and physical fitness were studied. Multiple linear regression models, including pairwise interaction terms involving total 25-OH-vitamin D, were fitted. The interacting effect of season and total 25-OH-vitamin D had a significant influence on physical fitness performance (spring and total 25-OH-vitamin D: ß 0.19, SE 0.07, p = 0.007; summer and total 25-OH-vitamin D: ß 0.10, SE 0.06, p = 0.11; autumn and total 25-OH-vitamin D: ß 0.18, SE 0.07, p = 0.01), whereas the main effect of total 25-OH-vitamin D alone was not significant (p = 0.30). Body fat percentage, recreational physical activity level, time spent per day gaming/TV-watching, smoking, and hemoglobin levels were also related to the physical fitness performance score. Future studies should further explore the role of seasonal-dependent effects of vitamin D on health.

## Introduction

Physical fitness in youth is a predictor of cardiovascular and musculoskeletal health in adulthood^1^. Vitamin D has been suggested to modulate physical fitness in adolescence^2^. The biological plausibility of this association relies on different possible mechanisms. Clinical observations indicate that a poor vitamin D status may lead to both peripheral myopathy and cardiomyopathy^3^. On the other hand, vitamin D promotes protein anabolism and muscle cell proliferation or differentiation. Similarly, vitamin D may influence myocardial contractility^4^ and has a well-recognized role in bone metabolism^5^. Finally, vitamin D has an important positive function in immune regulation and, therefore, in overall well-being^6,7^.

However, the few clinical studies focusing on the relationship between vitamin D and physical fitness among adolescents have provided inconsistent findings^8,9^, while recent observations on adults found an important association between serum vitamin D levels and physical fitness^10^. Most of the studies have not comprehensively explored the role of factors possibly associated with physical fitness, including dietary and sleeping behaviors, smoking and recreational activity attitudes, which might have an impact on lifestyle and health in adolescents^11–14^. On the other hand, increasing data indicate that vitamin D metabolism is more complex than initially thought and remains not fully understood^15^. Vitamin D values peak in summer and fall in winter^16^, and recent findings in youth have shown that the expression of genes modulated by vitamin D also shows a seasonal profile^17^. Although this hypothesis has never been investigated, it is tempting to assume an annual rhythm cycle in vitamin D action. This study aimed to investigate the effect of vitamin D on physical fitness performance across seasons in a large sample of male adolescents.

## Results

A total of 977 out of 4663 subjects who underwent medical examination volunteered to participate in the study. Anthropometrics, physical fitness performance, laboratory findings and lifestyle attitudes of the recruited subjects are given in Tables 1 and 2. Seventy-one (7.3%) out of 977 subjects had a body mass index ≥ 30.0 kg/m^2^, and 34 (3.5%) subjects had it ≤ 18.5 kg/m^2^. The physical fitness performance score ranged from 29 to 108, with a median of 74. The median level of total 25-hydroxy-vitamin D_3_ was 68 (interquartile range 55–82) nmol/L. No subject presented with levels of vitamin D_2_ > 5 nmol/L.

**Table 1:** Physical findings and laboratory characteristics of the 977 enrolled subjects.

N	977
**Physical findings**	
Body height (cm)	178.2 [173.0–181.9]
Body weight (kg)	72.6 [66.0–80.4]
Body mass index (kg/m^2^)	23.0 [21.0–25.1]
Body fat percentage (%)	17.7 [13.5–23.0]
Mid-upper arm circumference (cm)	27.0 [25.0–30.0]
Waist circumference (cm)	80.0 [74.5–87.0]
Waist to height ratio	0.45 [0.42–0.49]
Blood pressure (mm Hg)	132 [125–137]/75 [69–80]
Heart rate (beats/min)	74 [66–84]
**Laboratory**	
Hemoglobin (g/L)	155 [149–161]
Creatinine (µmol/L)	79 [73–86]
Total cholesterol (mmol/L)	3.9 [3.5–4.3]
High-density lipoprotein cholesterol (mmol/L)	1.2 [1.1.–1.4]
Total 25-hydroxy-vitamin D_3_ (nmol/L)	68 [55–82]

Data are given as median and interquartile range.

**Table 2:** Lifestyle characteristics of the 977 enrolled subjects.

N	977
**Recreational physical activity level, N (%)**	
Poor	203 (21)
Mild	182 (19)
Moderate	483 (49)
Intense	109 (11)
**Site of recreational physical activity, N (%)**	
Indoor (only)	139 (18)
Outdoor (only)	317 (41)
Both indoor and outdoor	318 (41)
**Frequency of gaming/TV-watching, N (%)**	
Never	237 (24)
1–2 weekly	40 (4)
3–4 weekly	269 (28)
5–6 weekly	114 (12)
Every day	317 (32)
**Time spent for gaming/TV-watching, N (%)**	
≤ 1 h per day	336 (45)
> 1–≤ 3 h per day	339 (46)
> 3–≤ 5 h per day	37 (5)
> 5 h per day	28 (4)
**Number of sleeping hours, N (%)**	
< 5 h per day	8 (1)
≥ 5–< 7 h per day	254 (26)
≥ 7–< 8 h per day	617 (63)
≥ 8–< 9 h per day	90 (9)
> 9 h per day	8 (1)
**Smoking, N (%)**	
Never	564 (58)
1–10 cigarettes per day	282 (29)
11–20 cigarettes per day	119 (12)
> 20 cigarettes per day	12 (1)
**Frequency of alcohol consumption, N (%)**	
Never	184 (19)
1 weekly	366 (38)
2 weekly	269 (28)
3–4 weekly	105 (11)
5–6 weekly	42 (4)
Every day	11 (0.1)
**Frequency of soda consumption, N (%)**	
Never	168 (17)
1–3 weekly	427 (44)
4–6 weekly	224 (23)
Every day	158 (16)
**Frequency of snack consumption, N (%)**	
Never	163 (17)
1–3 weekly	466 (48)
4–6 weekly	244 (25)
Every day	104 (10)
**Frequency of fruit consumption, N (%)**	
Never	56 (6)
1–3 weekly	212 (22)
4–6 weekly	259 (27)
Every day	450 (46)

Data are given as absolute frequency and percentage.

The results of univariate linear regression models are reported in the “Supplementary Materials”. Table 3 presents the results from a multiple regression analysis. After model selection based on the AIC and clinical plausibility, in the model including only the main-effects *body fat percentage* (ß − 0.94, SE 0.07, p < 0.0001), *recreational physical activity level* (moderate: ß 6.02, SE 0.94, p ≤ 0.0001; intense: ß 9.11, SE 1.35, p ≤ 0.0001), *site of recreational physical activity* (both indoor and outdoor: ß 2.37, SE 1.10, p = 0.03), *time spent per day gaming/TV-watching* (≤ 1 h per day: ß − 0.03, SE 1.03, p = 0.97; > 1–≤ 3 h per day: ß − 1.97, SE 1.04, p = 0.06; > 3–≤ 5 h per day: ß − 5.72, SE 2.35, p = 0.02; > 5 h per day: ß − 6.72, SE 3.09, p = 0.03), *smoking* (1–10 cigarettes per day: ß − 0.96, SE 0.89, p = 0.28; 11–20 cigarettes per day: ß − 2.46, SE 1.32, p = 0.06; > 20 cigarettes per day: ß − 10.27, SE 4.38, p = 0.02), and *hemoglobin* (ß 0.10, SE 0.04, p = 0.02), and *total 25-OH-vitamin D* (ß 0.07, SE 0.02, p = 0.001) were related to the physical fitness performance score, whereas seasonality was not significantly related to it. After inspection of models including *total 25-OH-vitamin D*-related interaction terms*,* the final model selected (likelihood ratio test on interaction term: p = 0.03) showed that the interacting effect of *season* and *total 25-OH-vitamin D* (spring and total 25-OH-vitamin D: ß 0.19, SE 0.07, p = 0.007; summer and total 25-OH-vitamin D: ß 0.10, SE 0.06, p = 0.11; autumn and total 25-OH-vitamin D: ß 0.18, SE 0.07, p = 0.01) had a significant effect on physical fitness performance, whereas the main effect of *total 25-OH-vitamin D* became nonsignificant (p = 0.30).

**Table 3:** Results of multiple regression analyses including main effects only (left part) and main effects together with an interaction term between season and total 25-OH-vitamin D (right part) (N = 977).

	Main-effects model			Interaction model		
	β	SE	p-value	β	SE	p-value
Body fat percentage (%)	− 0.94	0.07	< 0.0001	− 0.95	0.07	< 0.0001
Mid-upper arm circumference	0.26	0.14	0.06	0.29	0.14	0.04
**Recreational physical activity level**						
Moderate	6.02	0.94	< 0.0001	5.82	0.94	< 0.0001
Intense	9.11	1.35	< 0.0001	8.89	1.35	< 0.0001
**Site of recreational physical activity**						
Both indoor and outdoor	2.37	1.10	0.03	2.38	1.10	0.03
Outdoor (only)	0.68	1.10	0.54	0.6	1.10	0.59
**Time spent for gaming/TV watching**						
≤ 1 h per day	− 0.03	1.03	0.97	− 0.06	1.02	0.95
> 1–≤ 3 h per day	− 1.97	1.04	0.06	− 2.11	1.04	0.04
> 3–≤ 5 h per day	− 5.72	2.35	0.02	− 6.12	2.34	0.009
> 5 h per day	− 6.72	3.09	0.03	− 7.04	3.08	0.02
**Smoking**						
1–10 cigarettes per day	− 0.96	0.89	0.29	− 1.10	0.88	0.21
11–20 cigarettes per day	− 2.46	1.32	0.06	− 2.35	1.31	0.07
> 20 cigarettes per day	− 10.27	4.38	0.02	− 9.57	4.37	0.03
**Laboratory**						
Hemoglobin (g/L)	0.10	0.04	0.02	0.10	0.04	0.02
Creatinine (µmol/L)	0.06	0.03	0.10	0.06	0.03	0.72
Total 25-OH-vitamin D	0.07	0.02	0.001	− 0.06	0.06	0.30
**Season**						
Spring	1.45	1.18	0.22	− 9.62	4.24	0.02
Summer	− 1.77	1.36	0.20	− 6.90	4.36	0.11
Autumn	− 0.11	1.42	0.94	− 11.21	4.83	0.02
**Season and total 25-OH-vitamin D interaction**						
Spring: total 25-OH-vitamin D				0.19	0.07	0.007
Summer: total 25-OH-vitamin D				0.10	0.06	0.11
Autumn: total 25-OH-vitamin D				0.18	0.07	0.01

The main-effects model was selected based on the best Akaike Information Criterion. The original model included the following independent variables: body fat percentage, mid-upper arm circumference, season, site and level of recreational physical activity, frequency of alcohol consumption, frequency of soda consumption, frequency of snack consumption, frequency of fruit consumption, smoking, adequacy of sleeping hours, frequency and hours spent per day for gaming/TV watching, hemoglobin, creatinine and total 25-OH-vitamin D. Physical fitness performance was the dependent variable. The Multiple R-squared derived from the main-effects model was 0.39.

The interaction model was selected starting from the best main-effects model on the left part of the table and adding each interaction term involving total 25-OH-vitamin D one at a time. The best interaction model was selected based on the likelihood ratio test statistics comparing models including versus excluding each interaction term. After selection, the final model included the interaction term between season and total 25-OH-vitamin D. The interaction between categories of the two variables was indicated with “:” The Multiple R-squared derived from the interaction model was 0.40.

*β* beta-coefficient, *SE* standard error.

**Figure 1:**
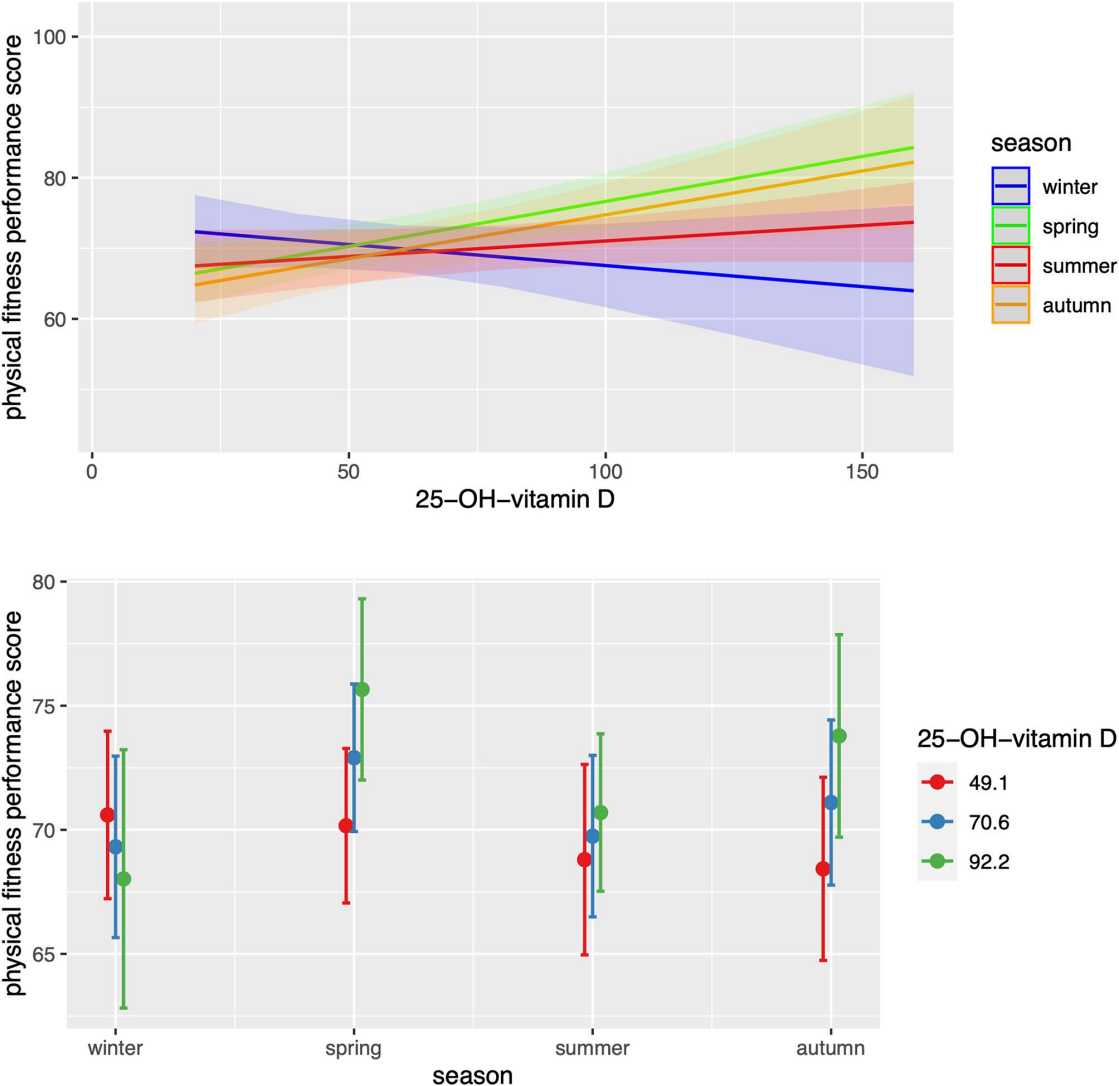
Predicted physical fitness performance scores across different seasons and total 25-OH-Vitamin D values. Results were obtained from the final multiple regression model selected, which included the interaction term between season and total 25-OH-Vitamin D values, as well as the following main effects: body fat percentage, mid-upper arm circumference, season, recreational physical activity level, site of recreational physical activity, smoking, time spent per day for gaming/TV watching, hemoglobin, creatinine, and total 25-OH-vitamin D. All the model predictors that are not specified in the interaction term were held constant. Upper panel: season-specific regression lines and 95% confidence intervals for increasing values of total 25-OH-Vitamin D on the x-axis. Lower panel: season-specific predicted physical fitness performance scores and 95% confidence intervals for three combinations of total 25-OH-Vitamin D values representing the mean (70.6 nmol/L, blue-center) minus or plus 1 standard deviation [49.1 nmol/L (red-left) and 92.2 nmol/L (green-right), respectively].

Figure 1 shows the interaction effect of total 25-OH-vitamin D and season on predicted physical fitness performance scores among participants. In the upper panel, season-specific regression lines showed a different trend for increasing values of total 25-OH-vitamin D. Compared to that for winter (with an intercept equal to the overall mean of the reference category and a slope of − 0.04), the remaining season-specific trajectories had: (1) lower intercepts, with autumn season showing the lowest intercept, significantly different from zero and from the other ones and positive slopes, with spring and autumn showing similar slopes, both significantly different from zero. Confidence bands for winter also had minimal overlap with spring and autumn, although confidence intervals became wider for the highest values of total 25-OH-vitamin D. Similarly, in the lower panel, within the same season, predicted physical fitness performance scores showed the previously described trend across three selected values of total 25-OH-vitamin D: (1) autumn and spring showed the highest (positive) differences at increasing total 25-OH-vitamin D values compared to that in winter (negative trend), with no material changes (although positive) in summer; (2) for a fixed season, the overlapping of confidence intervals was minimum for autumn and then spring. Furthermore, for a fixed value of total 25-OH-vitamin D, the predicted physical fitness performance scores diverged more in autumn and spring than in the other seasons. In conclusion, while in spring and autumn, a positive and significant linear relationship between vitamin D and physical fitness was present (with autumn having a significantly lower intercept), summer was not materially different from winter in either the intercept or the slope of the regression model.

## Discussion

The results of this study conducted in a sample of approximately 1000 male late adolescents support previous evidence that body fat, time spent gaming/TV-watching, and smoking are inversely related to physical fitness performance. Furthermore, they confirm that recreational physical activity and hemoglobin are positively associated with physical fitness performance. The novelty of this study is that after adjustment for the previously mentioned factors, vitamin D values modulated physical performance in interaction with seasonality: compared to winter, spring or autumn provides a different effect of vitamin D on physical fitness performance. In those seasons with the mildest climate, for higher values of total vitamin D, the physical fitness score significantly increased. A significantly lower intercept was also found in autumn.

Previous studies have considered the independent role of vitamin D in physical fitness in adolescents, providing inconsistent results. The HEalthy Lifestyle in Europe by Nutrition in Adolescence (HELENA) study found that muscular strength was not associated with vitamin D values in ~ 350 male adolescents with a mean age of 15 years^9^. A further large study including ~ 500 male adolescents living in Northern Ireland found that subjects 15 years of age in the highest tertile of vitamin D concentration had higher muscle strength than those in the lowest tertile^8^. The same study did not detect any difference in children 12 years of age. However, the mentioned studies did not adjust the results for confounders, such as recreational physical activity and hemoglobin, which significantly contribute to physical fitness^18–20^. Moreover, none of these studies tested for the possible synergic role of vitamin D with single recognized determinants of physical fitness performance. Among these determinants, our analysis suggests that seasonality has a key role in modulating the effect of vitamin D. Indeed, it is well known that seasonal changes impact many aspects of adolescents’ health, including body composition, cardiovascular system functioning, and general well-being^21–24^. A seasonal variability in vitamin D receptors (which is lowest in the winter season) and gene expression associated with immune function has also been recently demonstrated^17^. Vitamin D acts on all these factors, which, in turn, determine physical fitness, including cardiorespiratory fitness and muscular strength^25–27^.

The positive association of hemoglobin, body fat percentage, mid-upper arm circumference with physical performance is not unexpected. Oxygen transport to muscle cells strongly depends on hemoglobin, which carries almost all of the oxygen in the bloodstream^28^. Body fat percentage and mid-upper arm circumference are widely used as indices of body composition and nutritional status^29^. These variables were associated in opposite directions to physical fitness. The time spent gaming/TV-watching and the number of cigarettes consumed per day were inversely associated with physical fitness performance in our study. These findings are in line with other recent observations^30,31^: screen time, such as gaming or TV-watching, is negatively associated with physical performance in youth, even after adjusting for physical activity. Similarly, smoking impairs peripheral oxygen perfusion by several mechanisms affecting the blood, vessels, heart, and lung^32^. In turn, increased physical activity might result in a reduced tendency to smoke among adolescents^33,34^.

After adjustment for confounders, we did not find any association between physical performance and adequacy of sleeping hours. Clinical studies on adults have shown a mutual relationship between sleep and physical fitness. This association would be mediated by the different levels of physical activity (e.g., subjects with better sleep would present less sleepiness during the day and be more active)^35^. We included physical activity level and sleep as independent variables in the regression model, and we also tested the effect of their interaction on physical fitness performance. When we added the physical activity level to the univariate model including adequacy of sleeping hours, the significant association of sleeping behavior with physical fitness performance disappeared; however, the interaction between sleeping and physical activity level was nonsignificant. Therefore, our data do not allow us to derive any conclusions on the direct or indirect effect of sleep on physical fitness performance. Finally, we did not detect any association between the frequency of alcohol, soda, snack and fruit consumption and physical fitness performance. At least two possible explanations may be given. First, it is possible that such factors could become relevant only when persisting for longer time periods. Second, some data suggest that young individuals with unhealthy behaviors tend to compensate for other healthy attitudes (e.g., eating fewer calories or being more active)^36,37^.

This study has many potential clinical and research implications. First, our data support the recently given advice for adults that subjects with suboptimal vitamin D status should be investigated for their physical fitness status^10^. In addition, future studies investigating the role of vitamin D in health should consider the concurrent role of physiological variability occurring with seasonal changes. Finally, the possible synergic effect of seasonality should be considered when analyzing the results of vitamin D supplementation on physical fitness.

This study has strengths and limitations. Three main strengths are worth emphasizing. First, the large number of healthy subjects enrolled represents, to the best of our knowledge, the hitherto most numerous sample of male adolescents studied in relation to physical fitness performance and vitamin D. Second, the included population was unselected with respect to health habits. Third, we considered a number of potential confounders, including body composition, lifestyle factors and laboratory findings. The main limitation of this study is that the results concerned males only. In addition, similar to studies conducted in adults^10^, our results are based in part on self-reported data, and despite that fact, we adjusted for as many potential confounders as we could, but a few other variables that might influence physical fitness (e.g., the intake of dairy products) were not investigated. The cross-sectional nature of the study does not allow us to longitudinally assess the role of vitamin D in physical fitness. Moreover, we did not collect data on sunlight exposure. However, the levels of 25-hydroxy-vitamin D_2_ were ≤ 5 nmol/L in participants, suggesting that vitamin D of plant origin, a potentially important modulator of vitamin D status, did not play a significant role in determining vitamin D levels. Finally, we did not investigate the potential role of genetic assets. Although the presence and possible role of vitamin D receptors on muscular tissues are still debated^38^, recent observations have suggested that vitamin D supplementation in women might have a different effect on muscular strength based on the vitamin D receptor genotype^39^.

In conclusion, the results of this study suggest that the action of vitamin D, a light-dependent hormone, on physical fitness follows seasonal rhythmicity. This action is stronger in seasons with a mild climate. Future studies should further explore the role of seasonal changes in vitamin D effects on health.

## Methods

This research is based on data from the “CENERI study”^16^. Briefly, the CENERI study aims to identify potential risk factors for developing chronic diseases in healthy male adolescents living in southern Switzerland. In Switzerland, all ostensibly male adolescents aged between 18 and 19 years undergo a medical examination before compulsory military service^40^.

### Participant enrollment and study procedures

For the purpose of this study, all apparently healthy male subjects attending the mentioned examination from January 2014 to December 2016 were eligible and consecutively enrolled. Subjects on supplementation with any form of vitamin D or managed with anticonvulsants, antiretroviral drugs, glucocorticoids, and antifungals or with any underlying endocrinologic, renal or metabolic disease potentially altering the metabolism of vitamin D were excluded.

For each subject who volunteered to participate, all data were prospectively collected by a trained nurse. Participants were asked to answer a structured questionnaire^16^ investigating current lifestyle (recreational physical activity, time spent gaming/TV-watching, smoking and sleeping behavior) and dietary (including alcohol, soda, snack and fruit consumption) habits. Body height, weight, arterial blood pressure, heart rate, and mid-upper arm and waist circumferences were measured. Body fat percentage was also assessed. Finally, blood samples were drawn.

### Lifestyle and dietary habits

Questions on lifestyle and dietary habits were as follows: (1) frequency of recreational physical activity (never, 1 weekly, 2–4 weekly, 5–6 weekly, every day), (2) duration of recreational physical activity session (≤ 1 h, > 1–≤ 2 h, > 2–≤ 3 h, > 3 h), (3) site of recreational physical activity (indoor only, outdoor only, both indoor and outdoor), (4) frequency of gaming/TV-watching (never, 1–2 weekly, 3–4 weekly, 5–6 weekly, every day), (5) average time spent for any session of gaming/TV-watching (≤ 1 h, > 1–≤ 3 h, > 3–≤ 5 h, > 5 h), (6) smoking (never, 1–10 cigarettes per day, 11–20 cigarettes per day, > 20 cigarettes per day), (7) alcohol consumption (never, 1 weekly, 2 weekly, 3–4 weekly, 5–6 weekly, every day), (8) soda consumption (never, 1–3 weekly, 4–6 weekly, every day), (9) snack consumption (never, 1–3 weekly, 4–6 weekly, every day), and (10) fruit consumption (never, 1–3 weekly, 4–6 weekly, every day).

### Anthropometric data, blood pressure and heart rate

Subjects were weighed on a calibrated platform scale wearing light clothes only. Weight was rounded off to the nearest 0.1 kg. Standing height was measured barefooted to the nearest 0.1 cm. Body height and weight were used to calculate the body mass index. Mid-upper arm circumference was measured midway from the olecranon and the acromion in the nondominant arm to the nearest 0.1 cm. Waist circumference was measured with a nonstretching tape placed around the abdomen at the iliac crest to the nearest 0.5 cm. Waist circumference and height were used to calculate the waist-to-height ratio. Body fat percentage was measured by a validated bioimpedance analysis device (Omron BF306, Omron Healthcare Europe BV, Hoofddorp, the Netherlands)^41,42^. Blood pressure and heart rate were measured by a validated oscillometric automatic device (Microlife BPA6PC, Microlife Corp., Taipei, Taiwan). If the systolic or diastolic blood pressure was ≥ 140 mmHg or ≥ 90 mmHg, respectively, three additional measurements were obtained 15 s apart, and the mean value was considered.

### Physical fitness assessment

Within the abovementioned examination, conscripts undertook a validated physical test to assess physical fitness performance^43–46^. This test, described elsewhere in detail^44,45^, evaluates speed and endurance (to assess aerobic endurance capacity); the strength of the legs, arms and trunk; and balance, providing a global score of the physical fitness performance that ranges from 0 to 125^43–46^.

### Laboratory analysis

Advia technology was used for the determination of hemoglobin, an enzyme assay was used for the determination of creatinine, total cholesterol and high-density lipoprotein cholesterol in serum. Hemoglobin (cyanide-free colorimetry assay) was determined in whole blood, and creatinine (enzyme assay), total cholesterol (enzyme assay), high-density lipoprotein cholesterol (enzyme assay) and total 25-OH vitamin D were determined in serum. For the determination of the total 25-OH vitamin D concentration, an Abbott chemiluminescent microparticle immunoassay was used, which measures both 25-OH vitamin D_2_ and 25-OH vitamin D_3_^47^. The precision and sensitivity of the assay have been recently reported^16,47^. The reliability and accuracy of the assay were assessed both in the Vitamin D External Quality Assessment Scheme and in the Vitamin D Standardization Program^48,49^. All laboratory analyses were performed in the same accredited laboratory (Viollier, Basel, Switzerland) using an Architect CI8200 (Abbott) analyzer.

### Data analysis

#### Descriptive statistics

The distribution of the continuous variables was visually checked by means of histograms and tested by the Kolmogorov–Smirnov test. Since most variables were not normally distributed, we decided to report continuous data as medians and interquartile ranges. Categorical variables are presented as absolute values and relative frequencies. The recreational physical activity was evaluated considering both the frequency and duration of the activity, which were scored as follows: (a) for the frequency, never = 0; 1 weekly = 1; 2–4 weekly = 2; 5–6 weekly = 3; and every day = 4; and (b) for the duration, ≤ 1 h = 1, > 1–≤ 2 h = 2, > 2–≤ 3 h = 3, and > 3 h = 4. The recreational physical activity level was subsequently assessed after multiplying the frequency and duration scores as follows: 0 = poor, 1–2 = mild, 4–7 = moderate, and ≥ 8 = intense.

#### Inferential statistics

To investigate potential determinants of physical fitness performance (main outcome), we fitted a series of simple (i.e., one independent variable at a time) and multiple (i.e., two or more independent variables simultaneously included in the same model) regression models. In detail, the fitted ordinary least squares regression models included the physical fitness performance score (continuous) as the dependent variable and the following independent variables: body fat percentage, mid-upper arm circumference, astronomical season, site and level of recreational physical activity, frequency of alcohol consumption, frequency of soda consumption, frequency of snack consumption, frequency of fruit consumption, smoking, adequacy of sleeping hours (sufficient ≥ 7 or insufficient < 7 h)^50^, frequency and hours spent per session of gaming/TV-watching, hemoglobin, creatinine and total 25-OH-vitamin D. The best multiple (main-effects) model was selected using the Akaike Information Criterion (AIC) combined with clinical plausibility. Given that our main focus was on vitamin D, we also examined all the regression models presenting pairwise interaction terms, including total 25-OH-vitamin D, as one of the two variables. In detail, we added one interaction term at a time to the AIC-selected main-effects model; evidence of a potential interaction effect was explored with the likelihood ratio test comparing AIC-selected main-effects models including versus excluding each interaction term. Significance was assumed when p < 0.05 for the main effects and when p < 0.1 for the interaction effects. The statistical analysis was performed using the R software, version 3.5.3 (2019-03-11).

### Ethics approval

The study was approved by the Institutional Ethical Committee of Southern Switzerland (RIF CE 2775), and informed written consent was obtained from all participants. The study was performed in accordance with the principles of the Declaration of Helsinki and its later amendments.

## Supplementary Information

Supplementary Table 1.

## Abbreviations

AIC: Akaike Information Criterion
SE: Standard error
