# Supplementary material for: Seasonal variability of the vitamin D effect on physical fitness in adolescents
Source: Sci Rep. 2021 Jan 8;11:182. doi: 10.1038/s41598-020-80511-x (PMC7794427; doi:10.1038/s41598-020-80511-x)
Supplement: Supplementary file 1 — Supplementary Table 1. [file 41598_2020_80511_MOESM1_ESM.docx]

**Supplementary materials**

**Seasonal variability of the vitamin D effect on physical fitness in adolescents**

**Gregorio P. Milani, Giacomo D. Simonetti, Valeria Edefonti, Sebastiano AG Lava, Carlo Agostoni, Maurus Curti, Andreas Stettbacher, Mario G Bianchetti, Franco Muggli**

**Supplemetary table 1.** Results of the simple univariate linear regression models

| **N** |  |  |  |
| --- | --- | --- | --- |
| **Physical findings** | 𝛽 | SE | p-value |
| Body fat percentage, % | -1.02 | 0.06 | <0.0001 |
| Mid-upper arm circumference, cm | -0.06 | 0.01 | <0.0001 |
| **Recreational physical activity level, N (%)** |  |  |  |
| Mild | 2.90 | 1.28 | 0.02 |
| Moderate | 11.69 | 1.05 | <0.0001 |
| Intense | 16.87 | 1.49 | <0.0001 |
| **Site of recreational physical activity, N (%)** |  |  |  |
| Outdoor (only) | 4.76 | 1.35 | 0.0004 |
| Both indoor and outdoor | 0.19 | 1.35 | 0.88 |
| **Frequency of soda consumption, N (%)** |  |  |  |
| 1 - 3 weekly | -2.50 | 1.26 | 0.04 |
| 4 - 6 weekly | -3.40 | 1.41 | 0.02 |
| Every day | -3.71 | 1.53 | 0.02 |
| **Frequency of fruit consumption** |  |  |  |
| 1 - 3 weekly | 2.84 | 1.37 | 0.2 |
| 4 - 6 weekly | 1.79 | 2.03 | 0.4 |
| Every day | 4.83 | 1.95 | 0.02 |
| **Frequency of snack consumption** |  |  |  |
| 1-3 per week | -1.23 | 1.26 | 0.3 |
| 4-6 per week | -0.57 | 1.40 | 0.9 |
| Every day | -1.46 | 1.74 | 0.4 |
| **Frequency of gaming/TV watching, N (%)** |  |  |  |
| 1-2 weekly | 0.34 | 2.34 | 0.9 |
| 3-4 weekly | -2.94 | 1.22 | 0.02 |
| 5-6 weekly | 1.54 | 1.56 | 0.3 |
| Every day | -5.17 | 1.18 | <0.0001 |
| **Time spent for gaming/TV watching, N (%)** |  |  |  |
| >1 - ≤3 hours per day | -0.63 | 1.14 | 0.6 |
| >3 - ≤5 hours per day | -4.70 | 1.14 | <0.0001 |
| >5 hours per day | -10.5 | 4.44 | <0.0001 |
| **Number of sleeping hours, N (%)** |  |  |  |
| Insufficient | -2.29 | 0.99 | 0.02 |
| **Smoking, N (%)** |  |  |  |
| 1-10 cigarettes per day | -1.35 | 0.99 | 0.2 |
| 11-20 cigarettes per day | -5.19 | 1.38 | 0.0002 |
| >20 cigarettes per day | -12.9 | 3.99 | 0.002 |
| **Frequency of alcohol consumption, N (%)** |  |  |  |
| 1 weekly | 0.89 | 1.25 | 0.48 |
| 2 weekly | 1.67 | 1.32 | 0.21 |
| 3 - 4 weekly | 1.40 | 1.69 | 0.41 |
| 5 - 6 weekly | -2.32 | 2.36 | 0.33 |
| Every day | -2.86 | 4.29 | 0.51 |
| **Season** |  |  |  |
| Spring | 0.51 | 1.34 | 0.69 |
| Summer | -0.82 | 1.36 | 0.55 |
| Autumn | 10.14 | 1.58 | 0.93 |
| **Laboratory** |  |  |  |
| 25-hydroxy-vitamin D3, nmol/L | 0.13 | 0.02 | 0.0001 |
| Creatinine, mmol/L | 0.20 | 0.04 | <0.0001 |
